# Free odor identification engages domain-general cognitive abilities in old adults

**DOI:** 10.1093/chemse/bjaf049

**Published:** 2025-10-25

**Authors:** Thomas Hörberg, Jonas K Olofsson, Rohan Raj, Erika J Laukka, Maria Larsson

**Affiliations:** Department of Psychology, Sensory-Cognitive Interaction Lab/Gösta Ekman Laboratories, Stockholm University, Albanovägen 12, Stockholm 114 19, Sweden; Department of Psychology, Sensory-Cognitive Interaction Lab/Gösta Ekman Laboratories, Stockholm University, Albanovägen 12, Stockholm 114 19, Sweden; Department of Psychology, Sensory-Cognitive Interaction Lab/Gösta Ekman Laboratories, Stockholm University, Albanovägen 12, Stockholm 114 19, Sweden; Department of Neurobiology, Care Sciences and Society, Aging Research Center, Karolinska Institutet and Stockholm University, Tomtebodavägen 18a, Stockholm 171 65, Sweden; Stockholm Gerontology Research Center, Sveavägen 155, Stockholm 113 46, Sweden; Department of Psychology, Sensory-Cognitive Interaction Lab/Gösta Ekman Laboratories, Stockholm University, Albanovägen 12, Stockholm 114 19, Sweden

**Keywords:** odor naming, odor identification, cognitive abilities, cognitive aging

## Abstract

Naming common odors can be an exceptionally challenging task even for young and healthy individuals. Due to this difficulty, tests of cued odor identification (OID) are used instead of free odor identification in cognitive, neuropsychological, or aging research. Consequently, our understanding of the cognitive demands of free OID is limited. In this study, we analyze the demographic and cognitive factors that influence OID responses of old adults. We utilize a uniquely large dataset (*n* = 2,479) from a population-based sample of healthy, older Swedish adults (ages 58–102) who participated in free and cued OID using the 16-item Sniffin’ TOM test. The free OID naming responses were categorized as correct, misnamings, or omissions. The results revealed that omissions are surprisingly prevalent, constituting 66.4% of errors and accounting for 87.7% of the age-related differences in task performance. Additionally, we hypothesized that successful free OID would be more closely linked to nonolfactory cognitive abilities, such as verbal fluency, vocabulary, and episodic memory proficiency. This hypothesis was supported, as we found significant associations between free OID and these cognitive abilities, while cued OID identification only was associated with perceptual speed. Our findings suggest that the assessment of free OID may provide valuable insights into odor-based cognition, indicating a need for further research in this area.

## 1. Introduction

Naming common visual objects is for most people both easy and effortless, whereas naming failures belong to the clinical realm of neuropsychology, as it may indicate neurological disease effects on the language network (Kaplan et al. 2001). Odor naming (i.e. free odor identification; *free OID*), however, is different. Even for people that have a well-functioning sense of smell and for whom the odors are familiar, identifying a common odor source (e.g. *lemon*, *garlic* or *pine*) is surprisingly challenging in the absence of other cues. This phenomenon has been described as “common olfactory anomia” (Olofsson et al. 2013; Olofsson and Gottfried 2015). Already in the 1960s, the apparent intrinsic difficulty of the free OID task made researchers deem it impractical for olfactory diagnostics, and cued odor identification tasks (i.e. *cued OID*) were instead recommended for clinical use (Brain 1960; Sumner 1962). Free OID has since then only yielded a sparse interest as a cognitive research tool, whereas cued OID tests are the most widely used tests of olfaction (Doty et al. 1984b, 1996; Hummel et al. 1997). In the present work, we reconsider the utility of the free OID task, and propose that despite its difficulty, a free OID assessment may yield meaningful insights into cognition and the aging mind. We investigate free and cued OID responses generated by a large group of older participants (*n* = 2,479), focusing on how these tasks differ in terms of their associations with nonolfactory cognitive abilities.

A better understanding of the common olfactory anomia phenomenon in old adulthood may be accomplished by identifying the cognitive features that enable successful free OID. Successful free OID requires a perception of the odor quality, an association between the odor percept and stored semantic and/or episodic memory content that is indicative of its name, and the capacity to retrieve and verbalize that name. Evidence suggests that the common inability to name familiar smells may originate at either the perceptual level (Cain et al. 1998; Jönsson et al. 2005), the association phase (Herz and Engen 1996), or as a function of accumulated effects at perceptual, association and verbalization processing phases (Olofsson and Gottfried 2015). In most cultures studied, odors constitute an outlier as they are hard to consistently describe verbally (Majid et al. 2018; Olofsson and Pierzchajlo 2021). In aphasic patients with atrophy to their left ventrolateral prefrontal cortex (i.e. “Broca's area”), the ability to identify odors is more impaired than for visual objects, and this olfactory deficit proved to be exclusively correlated with the degree of atrophy in this region (Olofsson et al 2013). This observation suggests that free OID is more vulnerable to lesions in cortical areas subserving retrieval and verbalization processes, relative to visual object naming.

In the present work, we investigate how free and cued OID success of old adults is associated with cognitive and demographic factors. As free OID is considered more cognitively demanding, we hypothesize it is related to more cognitive abilities than cued OID (De Wijk and Cain 1994; Larsson 1997; Larsson et al. 2000; Larsson et al. 2004; Wehling et al. 2010). In cued OID, a set of response alternatives are presented verbally, one target in conjunction with a number of foils. Hence, the participant needs to match the perceived odor to a limited set of odor representations that are activated via semantic associations from the alternatives (Raj et al. 2023). In contrast, in free OID, the participant is asked to spontaneously retrieve the odor's lexical representation without any retrieval support, thereby requiring additional cognitive resources. In order to gain further insights into the cognitive demands underlying free OID, we expand on previous work by investigating free OID in terms of three different response outcomes; correct responses, incorrect responses (i.e. misnamings), and omissions (i.e. lack of responses).

Previous studies have mostly focused on cued OID or a combination of free and cued OID. In these studies, OID ability has been shown to be poorer in men than in women (Öberg et al. 2002; Wehling et al. 2016; Sorokowski et al. 2019; Xu et al. 2020) and to decline with age (Doty et al. 1984a; Hedner et al. 2010; Wehling et al. 2016; Xu et al. 2020), sometimes with a more pronounced decline for men than for women (Doty et al. 1984a; Cain and Stevens 1989; Murphy et al. 2002). Performance is usually better for well-educated people (Larsson et al. 2004; Fornazieri et al. 2019), and depends on the perceptual properties of the odors (Larsson et al. 2000; Lindroos et al. 2022). OID performance has also been associated with several cognitive abilities (as reviewed by Papazian and Pinto 2021; Challakere Ramaswamy and Schofield 2022) such as vocabulary/semantic memory (Larsson et al. 2000, 2004; Hedner et al. 2010; Finkel et al. 2011), episodic memory (Wehling et al. 2010, 2016; Finkel et al. 2011; and see the more recent meta-analysis by Jobin et al. 2023), executive functioning (Hedner et al. 2010), cognitive and perceptual speed (Larsson et al. 2004, 2005; Finkel et al. 2011), and verbal fluency (Larsson et al. 2005). Performance is also better for perceptually intense (Larsson et al. 2000) and more familiar (Wehling et al. 2010) odors. A shortcoming of some of these studies is that they have not drawn a clear distinction between cued and free OID (see, e.g. Cain 1979; Larsson et al. 2000; Ferdenzi et al. 2017). For example, Larsson et al. (2000) used a task where participants could pick an odor name from a list of 10 alternatives, but where they also were allowed to name the odor themselves. Their task thus involved both cued and free identification.

No prior studies have systematically compared free and cued OID performance of old adults in terms of their cognitive correlates. The hypothesis that free OID poses more, and different cognitive demands relative to cued OID has thus not yet been directly tested. To fill this gap, we will compare the cognitive demands of cued and free OID tasks by separately analyzing cognitive predictors of performance on these tasks. We will statistically control for the proficiency of the other odor task, such that in our analyses of free OID, we control for proficiency in cued OID, and in the analyses of cued OID, we control for free OID proficiency.

## 2. Materials and methods

### 2.1. Participants

Participants were from the Swedish National Study on Aging and Care in Kungsholmen (SNAC-K), a population-based study on aging and health that started in 2001. The original study sample consisted of 4,590 individuals who were randomly drawn from the population registry of Kungsholmen, a central area of Stockholm, Sweden. Of these, 3,363 took part in the baseline examination. Participants belonged to 11 prespecified age cohorts: 60, 66, 72, 78, 81, 84, 87, 90, 93, 96, and 99 years and older. Participants underwent comprehensive clinical, functional, and cognitive assessments. This examination involved a social interview and assessment of physical functioning; a clinical assessment of geriatric, neurological, and psychiatric information, and neuropsychological testing. In total, 2,848 participants completed the neuropsychological test battery, which also included olfactory assessment. Out of the 2,848 individuals who participated in the neuropsychological testing session, 2,569 completed the OID tests. Noncompleters did not perform the task due to self-reported anosmia (*n* = 95), odor oversensitivity (*n* = 17), asthma or allergies (*n* = 48), tiredness (*n* = 31), refusals (*n* = 36), or other reasons (*n* = 52; Larsson et al. 2016). We further excluded participants diagnosed with dementia (*n* = 60), Parkinson's disease (*n* = 20), schizophrenia (*n* = 9) and developmental disorders (*n* = 1), resulting in a final sample of 2,479 participants. The prevalence of depression was low (< 4%; Seubert et al. 2017) as the participants were sampled among the normal population and most of those with depression had mild depression. Participants with severe depression were less likely to participate in cognitive testing. The included participants had a mean age of 71.8 years, and an age range between 58 and 101 years, and 61.3% were women.

SNAC-K was approved by the Ethics Committee at Karolinska Institutet (Dnr 01-114) and was conducted according to the Declaration of Helsinki. All participants provided informed consent for participation.

### 2.2. Free and cued OID

**Fig. 1.**
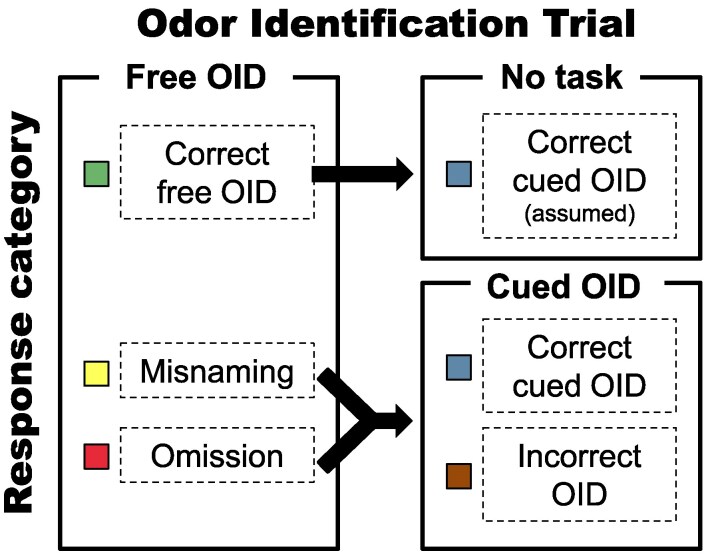
Graphical illustration of a trial in the OID task and the response outcomes. Participants were presented the odor and asked to freely identify it. If they were able to correctly identify it in the free OID task, the trial ended, and both the free and the cued OID response was categorized as correct. If they misnamed the odor or did not generate a name, the free OID task was categorized as a misnaming or an omission, respectively, and the trial continued with the cued OID task. If the participants correctly identified the odor among the response alternatives, the cued OID response was categorized as correct, otherwise it was categorized as incorrect.

Free and cued OID was assessed with the Sniffin’ TOM (Test of Odor Memory; Croy et al. 2015), a modified version of the 16-item Sniffin’ Sticks identification test (Hummel et al. 1997). During the test, the participant was asked to freely identify the odor directly following odor presentation. If the answer was incorrect or the participant was unable to provide a name, four labels (the target and three distractor labels) were presented in a forced-choice procedure. The 16 target odor names and their corresponding distractor labels are listed in Table 1, the testing protocol is illustrated in Fig. 1, and the top 20 most frequent misnamings per odor are shown in Supplementary Table S1.

**Table 1.** Target odors and the corresponding distractors in the OID test, originally collected by Larsson et al. (2016) and used in Raj et al. (2023).

Target odor	Distractor 1	Distractor 2	Distractor 3
**Apple**	Chocolate	Mint	Onion
**Banana**	Cherry	Coconut	Walnut
**Cinnamon**	Ham	Orange	Petrol
**Clove**	Banana	Cinnamon	Mustard
**Coffee**	Smoke	Tobacco	Wine
**Fish**	Bread	Cheese	Ham
**Garlic**	Apple	Carrot	Mint
**Leather**	Glue	Grass	Perfume
**Lemon**	Banana	Leather	Wine
**Licorice**	Cherry	Mint	Soap
**Mushroom**	Honey	Pine	Rum
**Peppermint**	Beer	Onion	Pine
**Petrol**	Blackberry	Pineapple	Strawberry
**Pineapple**	Chocolate	Onion	Vanilla
**Rose**	Cherry	Cucumber	Raspberry
**Turpentine**	Cheese	Mustard	Pear

All labels are translated from their Swedish originals.

### 2.3. Olfactory and cognitive assessment

The cognitive variables were collected as part of the neuropsychological testing session. They were selected for inclusion based on their explanatory relevance and previous empirical results (e.g. Larsson et al. 2004). The assessments are described briefly below. The complete study protocol is described in more detail in Laukka et al. (2013). The correlations between all participant variables are shown in Supplementary Table S2. The neuropsychological assessment that the olfactory testing was part of lasted about two hours. The olfactory assessment was administered either at the end or the middle of the testing session, according to two randomized test orders. Furthermore, three different test versions were used in order to minimize retest effects during follow-up (Larsson et al. 2016).

#### 2.3.1. Perceptual speed

Perceptual speed was assessed with two tests, a digit cancellation test (Zazzo 1974), and a pattern comparison test (Salthouse and Babcock 1991). In the digit cancellation test, participants go through eleven rows of random digits and are asked to cross out number four. The raw test score is the number of digits crossed out within 30 s. In the pattern comparison test, participants compare pairs of line-segment patterns on two pages, and are instructed to indicate whether the patterns are identical as quickly as possible. The raw test score is the number of correct classifications completed within 30 s per page, averaged across the two pages. The final perceptual speed score consisted of the average of the *z*-transformed digit cancellation and pattern comparison scores.

#### 2.3.2. Verbal fluency

Verbal fluency was measured with two letter fluency and two category fluency tasks. In letter fluency, participants generated as many words as possible that began with the letters F and A, respectively. The score on each task was the number of words that were generated in 60 s. In category fluency, participants generated as many words as possible that belonged to the categories animals and professions, respectively. Again, the score on each task was the number of correct words generated in 60 s. The final verbal fluency score was the average of the *z*-transformed scores of all four tasks.

#### 2.3.3. Vocabulary/semantic knowledge

Vocabulary was assessed with the 30-word vocabulary test SRB:1 (Dureman 1960; Nilsson et al. 1997). Each target word is presented with five other words, and the task is to identify the word that is synonymous to the target word. The raw test score is the number of synonyms that have been correctly identified within a time limit of seven min.

#### 2.3.4. Episodic memory

In line with previous research, episodic memory was evaluated with a free recall and a recognition test, using a word list of 16 semantically unrelated nouns (Salthouse 2010; Zahodne et al. 2011; Laukka et al. 2013; Sörman et al. 2017). Immediately after the words were presented, participants were given two min to orally recall as many words as possible. They then performed a self-paced recognition test, where they identified the original words from a list of the 16 target words intermixed with 16 distractor words. As a final score, we used the mean of the *z*-transformed free recall score, which was the total number of correctly recalled items, and the *z*-transformed recognition score, which was indexed by the number of hits minus the number of false alarms (In other words, the episodic memory score EM for the *i*:th participant is the average of participant *i*:s *z*-transformed free recall score FR and participant *i*:s *z*-transformed recognition score RC, EM*_i_* = (FR*_i_* + RC*_i_*)/2). We also tested the associations between OID performance and free recall and recognition separately.

#### 2.3.5. Executive function

Executive function was assessed with Trail Making Test B (TMT-B, see Pantzar et al. 2014). In the test, participants connect encircled numbers and letters in alternating order (1-A, 2-B, 3-C etc.). For participants with twelve or more correct connections, the performance score is the completion time of the task. In order to ensure that higher scores are indicative of superior performance, we reversed the scale by multiplying the original scores with −1.

#### 2.3.6. Free OID covariate

Each participant's general free OID ability was estimated with their free OID responses, calculated as the percentage of correct free OIDs of all odors that the participant at hand was exposed to.

#### 2.3.7. Cued OID covariate

In order to estimate each participant's general cued OID ability, we calculated a cued OID covariate for each participant. This score is the percentage of correct cued OIDs of all the odors that the participant was unable to correctly name. As such, this score is independent of the participants’ free OID ability. The free and cued OID covariates were used as control predictors in the statistical analyses.

### 2.4. Statistical analysis

All statistical analyses were conducted in R (R Core Team 2020). In order to draw conclusions regarding cognitive similarities and differences between free and cued OID, we analyzed free and cued OID performance with respect to demographic (age, sex, and number of years of education) and cognitive (perceptual speed, vocabulary, verbal fluency, episodic memory, and executive function as measured with TMT-B) factors.

Importantly, in our main analyses addressing our hypothesis we controlled for the proficiency of the other type of odor task; that is, models investigating free OID behavior included cued OID covariate as a control, and models of cued OID behavior comprised free OID covariate as a control. Hence, our models estimate the associations between demographic and cognitive variables with free and cued OID in concert, while accounting for the participants’ general ability to retrieve the semantic information in the respective retrieval format (free or cued OID). We also fitted models without such statistical adjustment. The results of these models are presented in Supplementary Materials Section S3, and referred to in the results section whenever they differ from those of the main models with the control covariates.

As some studies suggest that demographic variables, such as age and sex (e.g. Doty et al. 1984a; Cain and Stevens 1989; Murphy et al. 2002), interact with respect to olfactory performance (e.g. men showing a more pronounced age-related decline than women), we also included one-way interaction terms between demographic variables (i.e. sex × age, sex × education, and age × education).

For our main analyses, we used Bayesian multilevel logistic regression, predicting the change in the probability for a given outcome (e.g. correct free OID) as compared to the probability of other possible outcomes (e.g. misnaming), as a function of the participant and odor-specific variables listed above. *Correct free OIDs* involved all trials where participants correctly named the odor at hand and thus did not go through with the cued OID. *Misnamings* consisted of trials in which participants misnamed the odor during free OID, and *omissions* consisted of trials in which participants did not provide a name, independent of whether their subsequent cued OID attempt was correct. *Correct cued OIDs* involved all trials where participants either correctly named an odor during free OID or correctly identified the odor during subsequent cued OID. If a participant can freely identify an odor without cues, it is reasonable to assume that she would also be able to identify it among four different cues provided immediately thereafter (the odors were not presented a second time for cued OID due to time constraints). This assumption makes the free and cued OID responses independent from each other which leads to an unbiased statistical analysis (i.e. cued OID set is not affected by whether the free OID responses were correct or not). *Incorrect OIDs* consisted of all trials where the odor could not be identified. The response categories for free and cued OID and their relationship to the trial sequence are illustrated in Fig. 1.

All multilevel models were fitted with the R package brms (Bürkner 2017, 2018). Through the inclusion of random effects, multilevel regression controls for systematic effect differences between sampling units, such as participants or odors. In other words, the multilevel models can account for systematic differences in behavioral responses between participants (e.g. some participants being better at free OID but worse at cued OID than other participants) or odors (e.g. some odors being harder to name than others). All models therefore include by-participant and by-odor random intercepts.

For the main analyses of free OID data, we used both binomial and multinomial logistic regression. Multinomial modeling was used in order to compare differences between individual outcomes (e.g. comparing the probability of correct responses to the probability of omissions). We thus fitted three separate binomial models, investigating the change in the probability of (ⅰ) correct free OID (versus the probability of misnaming or omission), (ⅱ) misnaming (versus the probability of correct free OID or omission), and (ⅲ) omission (versus the probability of correct free OIDs and misnamings). We also fitted three multinomial models, one comparing the probability of correct free OID and the probability of misnamings to the probability of omissions, one comparing the probability of correct free OID and the probability of omissions to the probability of misnamings, and one comparing the probability of misnamings and the probability of omissions to the probability of correct free OID.

For cued OID data, we used one binomial model predicting the change in the probability of a correct cued OID response as compared to an incorrect OID response. For transparency, we also fitted a model of cued OID responses on data that only included trials with incorrect free OID responses (i.e. misnamings and omissions; see Supplementary Tables S15 and S16), allowing us to test whether our findings also holds when a correct free OID response is not assumed to entail a correct cued OID response.

In order to evaluate the models’ predictive ability, we performed posterior predictive checks of both the binomial and multinomial models. These analyses attested to high predictive accuracy of the models and are presented in Supplementary Materials Section S4.

All continuous variables were standardized by subtracting their sample mean and dividing by two standard deviations (following Gelman 2006: 57). We used standard weakly regularizing priors (Gelman 2006: 57; Gelman and Hill 2007; Stan Development Team 2017). For the intercept, we use a normal prior with a mean of zero and a standard deviation of 2.5. For the fixed effects, we use three degrees of freedom Student *t* priors with a mean of zero and a standard deviation of 2.5 units (following Gelman and Hill 2007). For random effect standard deviations, we use a Cauchy prior with location 0 and scale 2. All models were fitted with 12 chains with 1,000 warmup-samples and 4,000 post-warmup samples per chain, resulting in 48,000 posterior samples for each analysis.

All reported model effects are the maximum a posteriori (MAP) estimates of the effects’ posterior distributions. Inferences regarding effects are drawn on the basis of the MAP-based *P*-value (Mills and Parent 2014; Mills 2018). This is the estimated odds that an observed effect has against a null effect. It is the ratio of the density of the posterior distribution at the null value and the density of the posterior distribution at the MAP value. All bayesian statistics were derived with the R package BayestestR (Makowski et al. 2019).

It is important to note that the results of these analyses differ from the results of mere correlations between cued and free OID covariates, on the one hand, and the variables of interest, on the other (see Supplementary Table S3). The demographic and cognitive variables might very well be correlated with the free and cued OID covariates on their own, even in cases where they are not associated to the probability of individual OID responses when all other variables, as well as systematic differences between participants and odors, are controlled for. For transparency, in Supplementary Materials Section S5, we also present results of hierarchical regression models, similar to those of previous work (e.g. Larsson et al. 2004).

## 3. Results

### 3.1. Free OID responses across odors

**Fig. 2.**
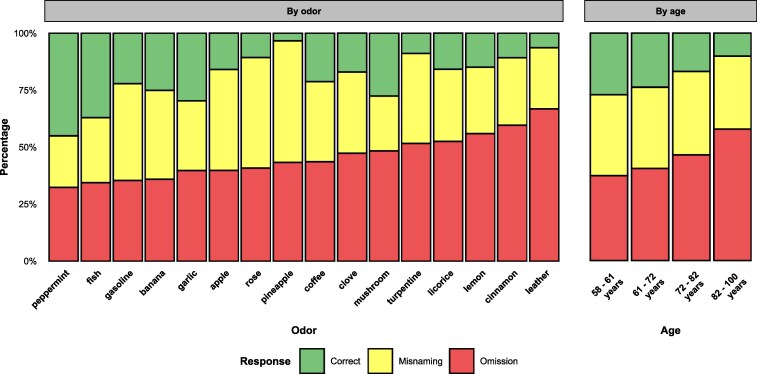
Odor-wise and age-wise percentages of correct responses, misnamings and omissions, ordered by the percentage of omissions, and by age, divided in first, second, third and fourth quantiles.

The frequency of correct free OID varied across odors, and the same was true for misnamings (wrong answers) and omissions (failure to generate any odor name; Fig. 2). Some odors, such as peppermint, are fairly easy to name (around 45% correct free OIDs) and others, such as leather, are much harder (only about 6% correct free OIDs). Notably, omissions are very common for all odors (range 32% to 67%) and increase with age. Whereas the mean percentage of omissions in the youngest age group (58 to 61 years) is about 37%, it is around 58% in the oldest age group (82 to 100 years). The percentage of misnamings (which also includes many near misses, e.g. *fruit* for pineapple odor), on the other hand, does not differ between the age groups. This suggests that old age is particularly associated with omission−a failure to generate any odor name.

### 3.2. Associations between OID responses and demographic as well as cognitive variables

**Fig. 3.**
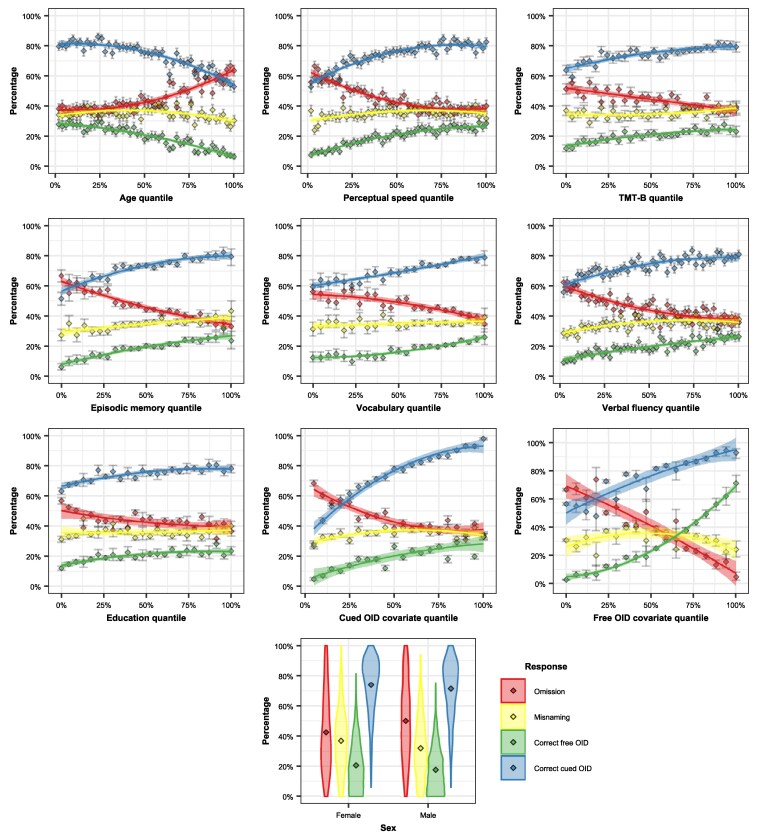
Percentage of older adults’ omissions, misnamings, correct free OIDs and correct cued OIDs as a function of participant variables age, education, perceptual speed, executive function (TMT-B), episodic memory, vocabulary, verbal fluency, cued OID covariate, free OID covariate and sex. For continuous participant variables, percentages are calculated within bins that are determined on the basis of the variable quantile. Variables with fewer unique values (e.g. cued OID covariate) contain fewer bins. Error bars illustrate 95% confidence intervals of the percentage of each bin, calculated on the basis of normal approximation.

We tested how free OID performance of old adults may depend on demographics and cognitive abilities (including cued OID and nonolfactory cognitive assessments). Figure 3 illustrates the percentage of omissions, misnamings, correct free OIDs and correct cued OIDs as a function of participant variables and OID scores. With the exception of sex, percentages are calculated within bins that are determined on the basis of the quantiles of each of the participant variables. Supplementary Table S3 shows Bayesian correlations between correct free OIDs, misnamings, omissions and correct cued OIDs, on the one hand, and all the quantitative participant variables, on the other. Table 2 provides an overview of the main findings from the Bayesian multilevel logistic regression models, presented below. Supplementary Tables S4–S23 present complete results of all multilevel models, both with and without total and free OID covariates as control predictors, and Supplementary Table S24 presents results of hierarchical regression modeling, performed on participant cued and free OID covariates.

**Table 2.** Summary of the main findings from the Bayesian multilevel logistic regression models, presented in the text.

Variable		Response			
		Correct free OID	Misnaming	Omission	Correct cued OID
**Demographic**	**Age**	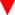	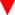 (vs. omission) 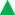 (vs. correct)	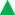	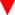
	**Education**	—	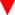 (vs. omission)	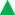 (vs. misnaming)	—
	**Sex (male)**	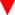	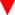	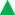	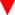
	**Age × Sex**	—	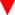	—	—
	**Age × Education**	—	—	—	—
	**Sex × Education**	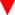 (vs. omission)	—	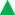 (vs. correct)	—
**Cognitive**	**Perceptual speed**	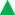	—	—	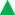
	**Vocabulary**	—	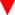	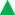	—
	**Verbal Fluency**	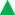 (vs. omission)	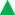	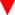	—
	**TMT-B**	—	—	—	—
	**Episodic Memory**	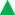	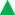	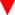	—
	**Recall**	—	—	—	—
	**Recognition**	—	—	—	—
**OID covariate**		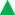	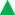 (vs. omission) 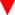 (vs. correct)	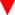	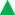

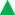
indexes a positive association between the variable and the response, and 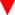 indexes a negative association between the variable and the response. If the association only is observed in comparison to one other response category, that reference category is presented in parentheses (e.g. omissions).

As illustrated in Fig. 3, OID behavior is strongly related to the participant variables. Both correct free OIDs and misnamings decrease with age and Executive function (TMT-B), whereas omissions increase dramatically in older age groups. Furthermore, both the rates of correct free OIDs and misnamings are positively associated with all other cognitive abilities (i.e. perceptual speed, executive functioning, episodic memory, verbal fluency and vocabulary). Cued OID ability also decreases with age, but it is higher in individuals with higher cognitive abilities (Fig. 3). Both free and cued OID abilities are also somewhat better for women than for men, as has been shown before in this dataset (Larsson et al. 2016).

**Fig. 4.**
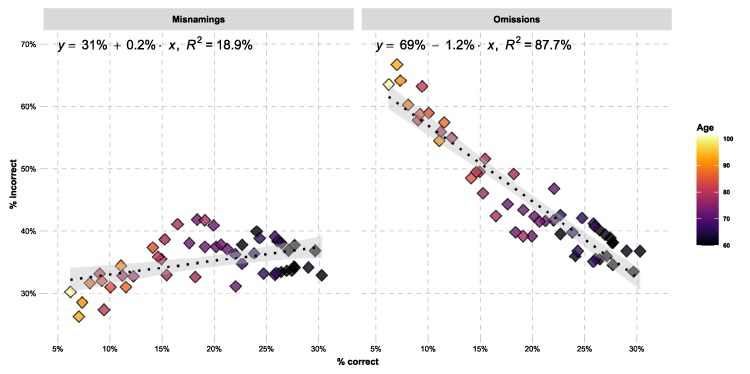
Percentage of misnamings and omissions as a function of percentage of correct free OIDs and age. All percentages are calculated within bins based on age quantiles and are thus “age-related”, showing the percentage within a particular age quantile. Omissions are more strongly related to age-related correct responses than misnamings.

In order to investigate which variables are associated with free and cued OID, we analyzed the data with Bayesian multilevel modeling. Emphasizing the difficulty of the free OID task, correct naming (i.e. correct free OID) of an odor is less frequent than either omitting the name (*β* = −1.06, SE = 0.27, *P* < 0.01) or misnaming it (*β* = −0.90, SE = 0.25, *P* < 0.01). Older participants are especially unlikely to provide the correct name (*β* = −0.62, SE = 0.08, *P* < 0.0001). The same pattern is found for cued OID success, which is lower in older age (*β* = −0.30, SE = 0.06, *P* < 0.0001). In free OID, this age-related impairment in correct responses is primarily related to a higher level of omissions (*β* = 0.43, SE = 0.09, *P* < 0.0001), rather than misnamings: The omission rate increases more rapidly with age than misnamings (*β* = 0.31, SE = 0.06, *P* < 0.0001). As illustrated in Fig. 4, the age-related difference in correct responses is much more strongly related to the age-related difference in omissions than to the age-related difference in misnamings. For each age-related percentage difference in correct responses there is a 1.2% difference in omission rates, but only a 0.2% difference in misnaming rates. Whereas age-related omission rates explains 87.7% of the variance in age-related correct response rates, age-related misnaming rates only explains 18.9% of that variance.

Bayesian models of both free and cued OID responses showed stronger associations between age and their respective OID responses (i.e. free and cued) when the OID score of the opposite response type was not controlled for (see Supplementary Materials Section S3). The association between age and cued OID responses, for example, was stronger in the model without free OID covariate included as a control predictor (*β* = −0.71, SE = 0.08, *P* < 0.0001) than in the model with free OID covariate (*β* = −0.30, SE = 0.06, *P* < 0.0001).

Women are better at free OID than men, both generally (*β* = 0.26, SE = 0.05, *P* < 0.0001), and in comparison to omissions (*β* = 0.48, SE = 0.07, *P* < 0.0001). Women also more frequently provide an incorrect name, rather than omitting the name completely (*β* = 0.41, SE = 0.06, *P* < 0.0001). Men further produce fewer incorrect responses with increasing age, as compared to women (*β* = −0.32, SE = 0.11, *P* < 0.05). Men thus appear to perform poorly on the free OID task because they often cannot come up with any odor name candidate. Women are also somewhat better at cued OID than men (*β* = 0.13, SE = 0.04, *P* < 0.01). This difference between women and men in cued OID performance was found to be even more pronounced when free OID covariate was not controlled for (*β* = −0.30, SE = 0.05, *P* < 0.0001), which is due to the fact that women are better in both tasks.

Interestingly, men with higher education perform somewhat *worse* in free OID than men with lower education, when free OID is analyzed in comparison to omissions (*β* = −0.32, SE = 0.12, *P* < 0.05). That is, highly educated men are more likely to omit the odor name than providing the correct one (*β* = 0.34, SE = 0.13, *P* < 0.05). When cued OID score is not controlled for, the opposite pattern is observed for women: women with higher education are more likely to provide the correct odor name, rather than omitting it (*β* = 0.25, SE = 0.09, *P* < 0.05).

As could be expected, cued OID covariate is the variable that predicts free OID success to the greatest extent. Participants with a high cued OID covariate are more likely to successfully name odors in general (*β* = 1.29, SE = 0.05, *P* < 0.0001), as compared to incorrect namings (*β* = 0.68, SE = 0.05, *P* < 0.0001), and to omissions (*β* = 1.05, SE = 0.07, *P* < 0.0001). High cued OID covariate is also associated with a higher probability for incorrect namings in comparison to omissions (*β* = 0.35, SE = 0.06, *P* < 0.0001). Conversely, and as would be expected, free OID covariate is associated with cued OID success (*β* = 1.29, SE = 0.05, *P* < 0.0001).

A higher percentage in correct free OIDs is associated with higher Perceptual Speed, and this association is substantially stronger when cued OID covariate is not controlled for (*β* = 0.27, SE = 0.07, *P* < 0.01) than when it is controlled for (*β* = 0.17, SE = 0.07, *P* < 0.05). The percentage of correct free OID is also high in individuals with high Episodic Memory scores, both in general (*β* = 0.14, SE = 0.05, *P* < 0.05), and in comparison to omissions (*β* = 0.27, SE = 0.07, *P* < 0.01). However, better Episodic Memory is also associated with a higher probability of misnaming an odor, rather than omitting the name (*β* = 0.18, SE = 0.06, *P* < 0.01). Similarly, better Verbal Fluency is associated with a higher probability for either correct free OIDs (*β* = 0.24, SE = 0.08, *P* < 0.0001) or misnamings (*β* = 0.25, SE = 0.07, *P* < 0.0001), in comparison to omissions. More verbally fluent participants are thus more likely to either correctly name or misname an odor, but less likely to omit the name completely. Vocabulary proficiency is, in contrast, not linked to a higher probability for correct free OID. Instead, participants with better vocabulary scores are more likely to *omit* an odor name, both in general (*β* = 0.26, SE = 0.08, *P* < 0.01), in comparison to providing the correct name (*β* = 0.23, SE = 0.09, *P* < 0.05), and in comparison to providing the incorrect name (*β* = 0.28, SE = 0.08, *P* < 0.01). In other words, participants with larger vocabularies are less likely to provide an incorrect odor name overall (*β* = −0.20, SE = 0.06, *P* < 0.01).

Additional analyses, shown in Supplementary Materials Section S3, that separated between free recall and recognition scores (i.e. the variables making up the composite score for Episodic Memory) did not find any association with neither word Recall nor word Recognition and free nor cued OID−only the combined Episodic Memory score was linked to olfactory performance.

Cued OID is not related to the same broad range of cognitive abilities as free OID. The only cognitive variable that is associated with a higher probability of cued OID success is Perceptual Speed (*β* = 0.16, SE = 0.06, *P* < 0.05), an association that is substantially stronger when free OID covariate is not controlled for (*β* = 0.28, SE = 0.07, *P* < 0.001). This means that even though the two olfactory tasks depend on the same perceptual functions, they pose quite different cognitive task demands.

## 4. Discussion

Free and cued odor identification (OID) is language-based, cognitive tasks used to assess olfactory ability, but their linguistic and cognitive underpinnings are relatively unknown, especially for free OID. Although free OID has been assumed to be more cognitively demanding than cued OID (Larsson 1997; Larsson et al. 2000, 2004) and might be more sensitive to perceptual properties of the odors (Lindroos et al. 2022), a systematic comparison between the two tasks on the level of individual responses has, to the best of our knowledge, not been conducted. We investigated to what extent these two tasks are associated with demographic and cognitive factors in a population of old adults. To further our understanding of the cognitive nature of free OID, our study is also the first to investigate both correct cued OID responses (i.e. correct namings), misnamings, and naming omissions in individual trials, while controlling for cued OID proficiency. In our analyses of cued OID, we focused on correct identifications in individual trials, while controlling for free OID proficiency.

Our findings confirm our hypothesis that old adults’ free OID draws on a wider range of cognitive abilities than cued OID. Whereas responses in cued OID were found to only be related to perceptual speed, responses in free OID were associated with perceptual speed, episodic memory, vocabulary/semantic knowledge, and verbal fluency. These findings are in line with the notion that free OID is more cognitively demanding (Larsson et al. 2000, 2004) and involves qualitatively different cognitive processing (Olofsson and Gottfried 2015). In the free OID task, the name of the odor needs to be retrieved from memory and verbalized. In contrast, cued OID, stored representations of the candidate odors (either in terms of valence or as an odor object, see e.g. Olofsson et al. 2012 for a discussion) need to be activated via semantic associations from the candidate odor names, and the participant must match the perceived odor to one of the stored odor representations (Raj et al. 2023). Apart from the linguistic decoding of the candidate names, cued OID can thus first and foremost be conceived as a perceptual-semantic matching task (e.g. Larsson et al. 2000; Hedner et al. 2010). We speculate that cued OID, as well as free OID, depend on cross-modal perceptual processing efficiency that is also required for perceptual speed task performance. Despite the shared reliance on perceptual speed, the stark observed differences in the cognitive correlates of cued and free OID should be considered when interpreting these two olfactory assessments.

Our findings differ from earlier studies that have found associations between cued OID and episodic memory (e.g. Wehling et al. 2010, 2016), vocabulary/semantic memory, (e.g. Larsson et al. 2004) as well as years of education (Larsson et al. 2004). However, the analyses in these studies were not conducted on the level of individual item responses, nor did they control for proficiency in free OID, which is needed to localize the cognitive underpinnings. In cued OID, participants might covertly generate candidate odor names directly when the odor stimulus is presented and then use those candidates to guide their response. The performance in the cued task will then be influenced by the ability to freely name an odor in assessment protocols which test cued and free OID concurrently. Several cognitive factors show high positive intercorrelations, especially among older adults where performance may exhibit a large interindividual variation, which may yield trivial correlations among cognitively demanding tasks (see Supplementary Table S2). Because the SNAC-K study uniquely includes both cued and free OID for the same set of odors in the same participants, we were able to assess and control for shared cognitive correlates among these two tasks.

In free OID analyses, omissions and misnamings are usually lumped together, and treated as incorrect responses. However, here we show that these two types of responses are differentially related to demographic and cognitive factors in old adults. An odor misnaming, as compared to an omission, is not random, but depends on a number of factors that might be informative. Omissions, but not misnamings, increase dramatically with age. Older participants are more likely to omit the odor name rather than providing an incorrect name, independently of the other cognitive factors evaluated in this study. The inclination to misname also differs between women and men: Women more frequently misname unknown odors, rather than omitting the name. We can only speculate about the causes of these demographic differences. The age difference might stem from a worse ability of older adults to retrieve lexical-semantic information (Raj et al. 2023). Aging may entail an impoverished ability to access more concrete candidate names that are high in specificity (e.g. *banana*). These difficulties, probably exacerbated by a noisier or weaker sensory input signal, may drive responses to less specific terms (e.g. *fruit*), or a complete omission. In line with this assumption, age-related decline in word recall typically affects concrete words more than abstract words (Peters and Daum 2008). Our results suggest that the ability to access and retrieve specific odor names decline strongly with age, which would explain why older participants make so many omission errors and, as we previously found, use more abstract odor descriptors (Hörberg et al. 2024).

Interestingly, we also found that men with higher education performed worse in free OID than less educated men. This finding is somewhat surprising as previous studies have found a positive relationship between level of education and olfactory performance in cued OID (Larsson et al. 2004; Fornazieri et al. 2019). Further analyses indicated that well-educated men more frequently omitted the odor name in comparison to providing the correct name, but not as compared to misnaming the odor. A speculation is that this patterns stems from a tendency among well-educated men to avoid guessing, which in this case might come at the expense of their overall performance. Other researchers have found similar results in other knowledge-based tasks (Merpert et al. 2018; Seo et al. 2021), adding to our interpretation that well-educated men are especially cautious when engaging with the inherently challenging task of naming odors.

Surprisingly, we also found that with increasing vocabulary proficiency, omissions increased relative to misnamings. We reason that people with more extensive vocabularies are more likely to omit the names of odors they do not know, rather than misnaming them, when other factors such as verbal fluency and episodic memory are taken into account. This finding could reflect a tendency of people with larger vocabularies to avoid guessing. People with better episodic memory and better verbal fluency are more likely to provide an incorrect name of unknown odors, rather than omitting their name. Given the evidence for strong interdependencies between episodic and semantic memory (e.g. Greenberg and Verfaellie 2010; Irish and Piguet 2013; Fang et al. 2018; Weidemann et al. 2019; De Brigard et al. 2022), this finding is not surprising. People with higher proficiency in semantic memory should be able to access more concrete odor candidate names than people with poorer semantic memory skills, resulting in a greater number of misnamings. The association between verbal fluency and misnaming rate is also expected. Persons who are more verbally fluent have a better ability to verbalize odor candidate names which could result in more misnamings as well as more correct free IDs as well as more misnamings, relative to omissions.

In order to account for proficiency in the other type of OID task, i.e. accounting for free OID proficiency in the analyses of cued OID performance and cued OID proficiency in analyses of free OID performance, cued and free OID covariates were included as control predictors in the multilevel models. For transparency, we also fitted models without these control predictors (see Supplementary Materials Section S3). Comparisons between these models showed that overall, the inclusion of these control scores mainly had a weakening effect on the associations between age, sex, and perceptual speed, on the one hand, and free OID and cued OID performance, on the other. These findings indicate that both OID tasks are influenced by age, sex, and perceptual speed, such that the association strengths between these variables and free and cued OID performance is weakened when proficiency in the other OID type is accounted for.

As our study focused on free and cued OID in old adulthood, our findings might not generalize to younger participants. In older age, individual variability tends to increase along with correlations among cognitive functions (Nyberg et al. 2012; Wilson et al. 2012; Hülür et al. 2015). The observed associations might thus not be constant across the adult lifespan. In order to provide some hints about how the observed associations between cognitive factors and OID performance might change with age, we performed additional analyses investigating the interactions between age and the cognitive variables (see Supplementary Tables S6, S9, S12, and S17). For cued OID, there were no significant interaction effects, indicating that associations between cognitive variables and cued OID performance do not change with age within the examined age range. For correct free OID responses, on the other hand, we found interactions between age and cognitive variables (TME-B and Perceptual Speed), that indicated that whereas executive functions became less important for successful free OID performance, the reliance on perceptual speed increases.

A potential limitation of the study might be that, in order to reduce testing time, each trial ended if participants correctly named the odor. We then made the assumption that if a participant was able to freely name an odor in the free OID task, she would also have selected that label from the list of four alternatives in the cued OID task. We performed control analyses on trials only including the cued OID task responses that showed that this assumption had no effect on the results (Supplementary Tables S13–S16).

In sum, free OID has been regarded as a very challenging task and evidence has been scarce regarding factors underlying successful free OID and how they may differ from cued OID. Here, we show that free OID engages a broad range of cognitive abilities which stands in sharp contrast to that observed for cued OID. Hence, the two types of olfactory assessment should not be regarded as equivalent measures. Failure to identify an odor in free OID might either result in a misnaming or a complete omission of the odor name. However, the choice between these two response modes was not random, as indicated by influences from both demographic and cognitive factors. Omissions rather than misnamings were observed as more frequent in aging. Future research might use these facets of OID proficiency to study specific effects of neurodegenerative disorders, but also when evaluating or enhancing olfactory capability in professional training programs within the perfumery or wine domains.

## Supplementary Material

bjaf049_Supplementary_Data

## Data Availability

The data that support the findings from this study are available from the Swedish National Study on Aging and Care in Kungsholmen database committee upon reasonable request.
