# Supplementary material for: Free odor identification engages domain-general cognitive abilities in old adults
Source: Chem Senses. 2025 Oct 25;50:bjaf049. doi: 10.1093/chemse/bjaf049 (PMC12603616; doi:10.1093/chemse/bjaf049)
Supplement: bjaf049_Supplementary_Data [file bjaf049_supplementary_data.pdf]

Free odor identification engages domain-general cognitive abilities in old adults - supplements

Thomas Hörberg[1], Jonas K Olofsson[1], Rohan Raj[1], Erika J Laukka[2,3] & Maria Larsson[1]

[1]Sensory-Cognitive Interaction Lab / Gösta Ekman Laboratory, Department of Psychology, Stockholm University, Stockholm, Sweden

[2]Aging Research Center, Department of Neurobiology, Care Sciences and Society, Karolinska Institutet and Stockholm University, Stockholm, Sweden

[3]Stockholm Gerontology Research Center, Stockholm, Sweden

1. Misnaming frequencies

Supplementary Table 1. The 20 most frequent misnamings of each odor.

**apple**	**N**	**banana**	**N**	**cinnamon**	**N**	**clove**	**N**
fruit	269	fruit	248	vanilla	67	spice	146
flower	99	apple	139	flower	65	cinnamon	77
candy	90	candy	70	perfume	55	hospital	45
perfume	74	pear	57	spice	51	allspice	43
raspberry	57	vanilla	57	soap	48	pepper	39
orange	51	sweet	35	weak	45	ginger	33
lemon	37	pineapple	32	fruit	19	cardamom	31
peach	36	strawberry	31	sweet	12	vanilla	24
pineapple	34	melon	28	bitter almond	11	flower	21
sweet	34	raspberry	25	detergent	11	ether	19
pear	33	flower	22	gingerbread	11	chemical	18
rose	32	perfume	20	bread	10	gingerbread	17
vanilla	30	good	19	chocolate	10	menthol	17
good	26	berry	17	good	10	mint	14
citrus	24	peach	16	cardamom	9	medicine	13
berry	23	chewing gum	15	ginger	9	vademecum	12
strawberry	21	chocolate	14	glue	9	leather	11
soap	19	orange	12	almond	8	strong	11
banana	14	mint	11	candy	8	cheesy	10
cherry	14	cherry	10	honey	8	fruit	10

**coffee**	**N**	**fish**	**N**	**garlic**	**N**	**gasoline**	**N**
chocolate	270	bad	82	cheesy	59	turpentine	85
spice	85	cheesy	57	spice	57	shoe polish	66
licorice	67	mushroom	51	bad	56	leather	60
cocoa	33	food	36	food	53	paint	55
bread	23	fermented baltic herring	26	cheese	27	tar	46
cinnamon	21	rotten	26	fermented baltic herring	18	oil	39
smoke	18	garbage	18	rotten	16	rubber	37
food	17	unpleasant	17	fish	15	chemical	36
burned	14	urine	16	food ooze	15	detergent	36
candy	14	cheese	15	unpleasant	14	solvent	32
sweet	13	sour	15	curry	13	color	30
caramel	12	stale	11	cabbage	11	white spirit	29
farin	12	steak ooze	11	cooking	11	kerosene	28
weak	10	crap	10	meat	11	smoke	21
leather	9	potato	10	mushroom	11	varnish	20
vanilla	9	onion	9	vegetable	11	exhaust	18
tar	8	spice	9	mustard	10	floor wax	17
gingerbread	7	yuk	9	burnt rubber	9	thinner	17
pastry	7	ammonia	8	nasty	9	asphalt	16
tobacco	7	weak	8	rubber	9	glue	16
**leather**	**N**	**lemon**	**N**	**licorice**	**N**	**mushroom**	**N**
soap	55	orange	74	spice	91	cheesy	36
perfume	43	fruit	70	flower	46	leather	36
chemical	27	spice	39	mint	42	bad	31
spice	27	flower	35	vanilla	39	spice	24
rubber	25	mint	32	candy	36	vegetable	21
detergent	21	candy	30	menthol	35	fish	19
petrol	20	perfume	25	perfume	31	earth	15
flower	18	soap	22	weak	31	onion	15
cinnamon	16	weak	18	eucalyptus	24	mold	14
wood	16	apple	14	peppermint	19	petrol	13
smoke	14	good	14	fruit	17	cheese	12
moth repellant	10	healthy	14	soap	17	flower	12
weak	10	sweet	13	violet	16	chemical	11
oil	9	chemical	12	breast caramel	15	food	10
cheesy	8	tart	12	fennel	15	forest	10
color	8	pine needle	11	sweet	15	fruit	10
dust	7	eucalyptus	10	orange	13	nasty	10
fruit	7	shoe polish	10	cardamom	12	moss	9
plastic	7	vanilla	10	cough drop	12	unpleasant	9
stale	7	lemon balm	9	cinnamon	10	cucumber	8

peppermint	N	pineapple	N	rose	N	turpentine	N
candy	54	fruit	311	perfume	445	shoe polish	124
candy cane	38	candy	128	soap	152	pine needle	61
eucalyptus	33	flower	87	flower	145	leather	54
spice	30	apple	74	lily of the valley	89	mint	43
vanilla	29	strawberry	73	lavender	51	petrol	34
fruit	23	perfume	61	jasmine	38	detergent	28
toothpaste	20	sweet	58	violet	38	chemical	27
lemon	19	soap	53	lilac	30	carrot	23
perfume	14	peach	42	skin cream	18	wood	21
vademecum	14	wild strawberry	42	detergent	16	mold	18
mint	12	orange	41	good	16	eucalyptus	17
soap	12	raspberry	41	eau de cologne	15	floor wax	16
chocolate	9	lemon	39	fruit	14	flower	16
petrol	9	cherry	35	sweet	13	menthol	16
sweet	9	vanilla	35	vanilla	13	spice	16
flower	8	pear	30	lemon	11	camphor	15
strong	8	citrus	29	ointment	11	glue	15
camphor	7	banana	25	shampoo	9	potato	14
banana	6	melon	25	spice	9	perfume	13
cough drop	6	berry	21	citrus	7	resin	13

2. Bayesian correlations between variables

Supplementary Table 2. Bayesian correlations between participant variables. All map-based *P*s < 0.0001.

	Age	Education	Verbal fluency	Vocabulary	TMT-B	Perceptual speed	Episodic memory	Recall	Recognition
Age	1.00	-0.34	-0.39	-0.31	0.35	-0.64	-0.30	-0.14	-0.39
Education	-0.34	1.00	0.43	0.43	-0.23	0.34	0.27	0.17	0.30
Verbal fluency	-0.39	0.43	1.00	0.56	-0.32	0.53	0.38	0.22	0.46
Vocabulary	-0.31	0.43	0.56	1.00	-0.34	0.39	0.34	0.23	0.37
TMT-B	0.35	-0.23	-0.33	-0.34	1.00	-0.39	-0.24	0.15	0.28
Perceptual speed	-0.63	0.34	0.53	0.39	-0.39	1.00	0.35	0.20	0.43
Episodic memory	-0.29	0.27	0.38	0.34	-0.24	0.35	1.00	0.89	0.84
Recall	-0.15	0.17	0.23	0.26	0.27	0.15	0.20	1.00	0.50
Recognition	-0.39	0.30	0.46	0.37	0.38	0.28	0.43	0.50	1.00

Supplementary Table 3. Bayesian correlations and MAP-based *p*-values for relationships between by-participant response percentages and participant variables. Note that the variable TMT-B measures response times and that a lower score thus entails better performance.

	**Omissions**		**Misnamings**		**Correct**		**Cued OID**	
	r	*p*	r	*p*	r	*p*	r	*p*
Age	0.31	<.0001	-0.07	<.0001	-0.43	<.0001	-0.45	<.0001
Education	-0.15	<.0001	0.05	.067	0.19	<.0001	0.20	<.0001
Verbal fluency	-0.25	<.0001	0.11	<.0001	0.28	<.0001	0.29	<.0001
Vocabulary	-0.14	<.0001	0.02	.669	0.21	<.0001	0.24	<.0001
TMT-B	0.13	<.0001	-0.03	.452	-0.18	<.0001	-0.21	<.0001
Perceptual speed	-0.27	<.0001	0.07	.003	0.36	<.0001	0.39	<.0001
Episodic memory	-0.22	<.0001	0.10	<.0001	0.24	<.0001	0.24	<.0001
Recall	-0.14	<.0001	0.08	<.0001	0.14	<.0001	0.15	<.0001
Recognition	-0.25	<.0001	0.10	<.0001	0.29	<.0001	0.29	<.0001

3. Multilevel Bayesian regression models

Supplementary Table 4. Results of Bayesian multilevel binomial regression models predicting correct free OID responses on the basis of demographic and cognitive variables, as well as cued OID covariates included as control. The effects of recall and recognition are also shown in the table. Note that these results come from models where the Episodic memory variable had been replaced with these variables.

Parameter	MAP	S.E.	CI lower	CI upper	p_{MAP}
Intercept	-1.72	0.25	-2.24	-1.23	<.0001
Age	-0.62	0.08	-0.77	-0.47	<.0001
Education	0.10	0.07	-0.02	0.24	.295
Sex (male)	-0.26	0.05	-0.37	-0.17	<.0001
Age × Sex	-0.15	0.11	-0.36	0.07	.404
Age × Education	0.15	0.11	-0.08	0.35	.473
Sex × Education	-0.25	0.10	-0.42	-0.04	.052
Perceptual speed	0.17	0.07	0.04	0.30	.039
Vocabulary	-0.08	0.06	-0.20	0.06	.475
Verbal Fluency	0.13	0.06	0.02	0.25	.095
TMT-B	-0.01	0.05	-0.12	0.09	.963
Episodic Memory	0.14	0.05	0.05	0.25	.021
Recall	0.10	0.06	-0.02	0.22	.256
Recognition	0.07	0.05	-0.04	0.18	.413
cued OID covariate	0.85	0.05	0.75	0.96	<.0001

Supplementary Table 5. Results of Bayesian multilevel binomial regression models predicting correct free OID responses on the basis of demographic and cognitive variables, without cued OID covariates as control. The effects of recall and recognition are also shown in the table. Note that these results come from models where the Episodic memory variable had been replaced with these variables.

Parameter	MAP	S.E.	CI lower	CI upper	p_{MAP}
Intercept	-1.69	0.25	-2.18	-1.18	<.0001
Age	-0.82	0.08	-0.98	-0.66	<.0001
Education	0.13	0.07	-0.01	0.27	.199
Sex (male)	-0.35	0.05	-0.45	-0.24	<.0001
Age × Sex	-0.17	0.12	-0.40	0.05	.354
Age × Education	0.18	0.11	-0.05	0.40	.296
Sex × Education	-0.26	0.10	-0.44	-0.05	.048
Perceptual speed	0.27	0.07	0.12	0.39	.001
Vocabulary	-0.04	0.07	-0.17	0.10	.843
Verbal Fluency	0.15	0.06	0.02	0.27	.082
TMT-B	-0.04	0.06	-0.14	0.08	.875
Episodic Memory	0.16	0.05	0.06	0.27	.011
Recall	0.14	0.06	0.01	0.26	.102
Recognition	0.06	0.06	-0.05	0.17	.576

Supplementary Table 6. Results of Bayesian multilevel binomial regression models predicting correct free OID responses on the basis of demographic and cognitive variables, cued OID covariates, and age interaction effects.

**Parameter**	**MAP**	**S.E.**	**Lower**	**Upper**	p_{MAP}
Intercept	-1.70	0.25	-2.21	-1.21	<.0001
Age	-0.67	0.08	-0.83	-0.52	<.0001
Sex (male)	-0.29	0.05	-0.38	-0.18	<.0001
Education	0.01	0.06	-0.10	0.12	.980
Perceptual speed	0.22	0.07	0.08	0.35	.010
Vocabulary	-0.09	0.07	-0.21	0.05	.448
Verbal Fluency	0.10	0.06	-0.02	0.23	.254
TMT-B	0.06	0.06	-0.06	0.16	.658
Episodic Memory	0.13	0.05	0.03	0.24	.053
Cued OID covariate	0.88	0.05	0.77	0.98	<.0001
Age × Sex	-0.07	0.11	-0.28	0.15	.863
Age × Education	0.15	0.12	-0.09	0.39	.475
Age × Perceptual speed	0.32	0.14	0.06	0.60	.058
Age × Vocabulary	-0.02	0.13	-0.29	0.23	.978
Age × Verbal Fluency	-0.09	0.14	-0.35	0.19	.850
Age × TMT-B	-0.32	0.10	-0.54	-0.13	.007
Age × Episodic Memory	-0.03	0.11	-0.26	0.19	.965
Age × Cued OID covariate	0.41	0.12	0.18	0.63	.002

Supplementary Table 7. Results of Bayesian multilevel binomial regression models predicting incorrect free OID responses on the basis of demographic and cognitive variables, as well as cued OID covariates included as control. The effects of recall and recognition are also shown in the table. Note that these results come from models where the Episodic memory variable had been replaced with these variables.

**Parameter**	**MAP**	**S.E.**	**CI lower**	**CI upper**	p_{MAP}
Intercept	-0.62	0.13	-0.89	-0.36	<.0001
Age	0.00	0.08	-0.15	0.15	1.000
Education	0.08	0.07	-0.05	0.22	.523
Sex (male)	-0.28	0.05	-0.38	-0.19	<.0001
Age × Sex	-0.32	0.11	-0.52	-0.10	.016
Age × Education	0.10	0.10	-0.11	0.30	.641
Sex × Education	0.02	0.10	-0.16	0.22	.973
Perceptual speed	0.00	0.07	-0.12	0.14	.998
Vocabulary	-0.20	0.06	-0.32	-0.07	.009
Verbal Fluency	0.16	0.06	0.03	0.27	.050
TMT-B	0.01	0.05	-0.09	0.12	.961
Episodic Memory	0.13	0.05	0.02	0.23	.049
Recall	0.03	0.06	-0.09	0.14	.889
Recognition	0.12	0.05	0.01	0.22	.128
cued OID covariate	0.10	0.05	0.00	0.19	.16

Supplementary Table 8. Results of Bayesian multilevel binomial regression models predicting incorrect free OID responses on the basis of demographic and cognitive variables, without cued OID covariates as control. The effects of recall and recognition are also shown in the table. Note that these results come from models where the Episodic memory variable had been replaced with these variables.

**Parameter**	**MAP**	**S.E.**	**CI lower**	**CI upper**	p_{MAP}
Intercept	-0.64	0.13	-0.89	-0.36	<.0001
Age	-0.02	0.08	-0.17	0.13	.972
Education	0.08	0.07	-0.05	0.22	.471
Sex (male)	-0.30	0.05	-0.39	-0.20	<.0001
Age × Sex	-0.31	0.11	-0.52	-0.11	.011
Age × Education	0.10	0.10	-0.09	0.31	.594
Sex × Education	0.03	0.10	-0.16	0.21	.936
Perceptual speed	0.01	0.07	-0.11	0.14	.967
Vocabulary	-0.19	0.06	-0.32	-0.07	.011
Verbal Fluency	0.15	0.06	0.04	0.27	.039
TMT-B	0.01	0.05	-0.09	0.11	.959
Episodic Memory	0.13	0.05	0.03	0.23	.051
Recall	0.03	0.06	-0.08	0.15	.871
Recognition	0.11	0.05	0.01	0.22	.126

Supplementary Table 9. Results of Bayesian multilevel binomial regression models predicting incorrect free OID responses on the basis of demographic and cognitive variables, cued OID covariates, and age interaction effects.

**Parameter**	**MAP**	**S.E.**	**Lower**	**Upper**	p_{MAP}
Intercept	-0.56	0.13	-0.82	-0.29	.001
Age	0.02	0.08	-0.12	0.18	.916
Sex (male)	-0.29	0.05	-0.38	-0.19	<.0001
Education	0.10	0.05	-0.01	0.20	.232
Perceptual speed	0.03	0.07	-0.10	0.16	.911
Vocabulary	-0.21	0.06	-0.33	-0.08	.007
Verbal Fluency	0.15	0.06	0.03	0.27	.063
TMT-B	-0.04	0.05	-0.15	0.07	.745
Episodic Memory	0.12	0.05	0.02	0.22	.066
Cued OID covariate	0.09	0.05	-0.01	0.18	.229
Age × Sex	-0.29	0.10	-0.50	-0.09	.021
Age × Education	-0.01	0.12	-0.25	0.21	.997
Age × Perceptual speed	0.26	0.13	0.01	0.51	.120
Age × Vocabulary	-0.10	0.13	-0.35	0.15	.800
Age × Verbal Fluency	-0.04	0.13	-0.28	0.23	.987
Age × TMT-B	0.04	0.10	-0.14	0.24	.844
Age × Episodic Memory	0.10	0.11	-0.11	0.31	.691
Age × Cued OID covariate	0.37	0.11	0.17	0.58	.002

Supplementary Table 10. Results of Bayesian multilevel binomial regression models predicting omission free OID responses on the basis of demographic and cognitive variables, as well as cued OID covariates included as control. The effects of recall and recognition are also shown in the table. Note that these results come from models where the Episodic memory variable had been replaced with these variables.

**Parameter**	**MAP**	**S.E.**	**CI lower**	**CI upper**	p_{MAP}
Intercept	-0.41	0.16	-0.74	-0.11	.037
Age	0.40	0.10	0.21	0.58	<.0001
Education	-0.20	0.09	-0.38	-0.03	.067
Sex (male)	0.45	0.06	0.33	0.57	<.0001
Age × Sex	0.32	0.13	0.04	0.56	.062
Age × Education	-0.08	0.13	-0.34	0.16	.812
Sex × Education	0.21	0.12	-0.02	0.46	.199
Perceptual speed	-0.09	0.08	-0.26	0.07	.510
Vocabulary	0.26	0.08	0.10	0.42	.004
Verbal Fluency	-0.28	0.08	-0.43	-0.13	.001
TMT-B	-0.01	0.06	-0.14	0.11	.974
Episodic Memory	-0.23	0.06	-0.34	-0.09	.004
Recall	-0.11	0.08	-0.26	0.04	.352
Recognition	-0.14	0.07	-0.27	-0.01	.107
cued OID covariate	-0.59	0.06	-0.72	-0.47	<.0001

Supplementary Table 11. Results of Bayesian multilevel binomial regression models predicting omission free OID responses on the basis of demographic and cognitive variables, without cued OID covariates as control. The effects of recall and recognition are also shown in the table. Note that these results come from models where the Episodic memory variable had been replaced with these variables.

**Parameter**	**MAP**	**S.E.**	**CI lower**	**CI upper**	p_{MAP}
Intercept	-0.43	0.16	-0.76	-0.13	.023
Age	0.55	0.10	0.35	0.73	<.0001
Education	-0.22	0.09	-0.40	-0.05	.053
Sex (male)	0.51	0.06	0.39	0.63	<.0001
Age × Sex	0.30	0.13	0.06	0.58	.055
Age × Education	-0.11	0.13	-0.38	0.14	.699
Sex × Education	0.12	0.12	-0.03	0.46	.217
Perceptual speed	-0.16	0.08	-0.32	0.01	.165
Vocabulary	0.23	0.08	0.08	0.39	.015
Verbal Fluency	-0.29	0.08	-0.44	-0.13	.001
TMT-B	0.00	0.07	-0.12	0.13	.997
Episodic Memory	-0.23	0.07	-0.36	-0.10	.002
Recall	-0.13	0.08	-0.28	0.01	.235
Recognition	-0.14	0.07	-0.27	0.00	.130

Supplementary Table 12. Results of Bayesian multilevel binomial regression models predicting omission free OID responses on the basis of demographic and cognitive variables, cued OID covariates, and age interaction effects.

**Parameter**	**MAP**	**S.E.**	**Lower**	**Upper**	$p_{\mathbf{MAP}}$
Intercept	-0.47	0.16	-0.80	-0.17	.017
Age	0.41	0.09	0.23	0.60	<.0001
Sex (male)	0.48	0.06	0.35	0.59	<.0001
Education	-0.11	0.07	-0.24	0.03	.296
Perceptual speed	-0.13	0.08	-0.29	0.04	.340
Vocabulary	0.27	0.08	0.11	0.43	.004
Verbal Fluency	-0.26	0.08	-0.41	-0.10	.005
TMT-B	0.00	0.07	-0.13	0.14	.999
Episodic Memory	-0.21	0.06	-0.34	-0.09	.005
Cued OID covariate	-0.57	0.06	-0.70	-0.46	<.0001
Age × Sex	0.23	0.13	-0.03	0.47	.214
Age × Education	-0.04	0.15	-0.32	0.26	.968
Age × Perceptual speed	-0.40	0.16	-0.71	-0.07	.061
Age × Vocabulary	0.08	0.16	-0.23	0.39	.884
Age × Verbal Fluency	0.10	0.16	-0.20	0.44	.801
Age × TMT-B	0.13	0.12	-0.10	0.38	.517
Age × Episodic Memory	-0.04	0.13	-0.30	0.22	.926
Age × Cued OID covariate	-0.34	0.13	-0.59	-0.07	.040

Supplementary Table 13. Results of Bayesian multilevel binomial regression models predicting correct cued OID responses on the basis of demographic and cognitive variables, as well as free OID covariates included as control. The effects of recall and recognition are also shown in the table. Note that these results come from models where the Episodic memory variable had been replaced with these variables.

**Parameter**	**MAP**	**S.E.**	**CI lower**	**CI upper**	$p_{\mathbf{MAP}}$
Intercept	1.34	0.22	0.91	1.77	<.0001
Age	-0.30	0.06	-0.42	-0.16	<.0001
Education	0.03	0.06	-0.09	0.14	.914
Sex (male)	-0.13	0.04	-0.21	-0.05	.009
Age × Sex	-0.03	0.09	-0.21	0.13	.892
Age × Education	0.06	0.09	-0.11	0.22	.783
Sex × Education	0.05	0.08	-0.11	0.20	.866
Perceptual speed	0.16	0.06	0.05	0.26	.017
Vocabulary	0.08	0.05	-0.02	0.18	.326
Verbal Fluency	-0.01	0.05	-0.11	0.09	.956
TMT-B	-0.03	0.04	-0.11	0.05	.806
Episodic Memory	0.01	0.04	-0.08	0.09	.974
Recall	0.05	0.05	-0.05	0.15	.588
Recognition	-0.03	0.04	-0.12	0.06	.791
free OID covariate	1.29	0.05	1.20	1.38	<.0001

Supplementary Table 14. Results of Bayesian multilevel binomial regression models predicting correct cued OID responses on the basis of demographic and cognitive variables, without free OID covariates as control. The effects of recall and recognition are also shown in the table. Note that these results come from models where the Episodic memory variable had been replaced with these variables.

**Parameter**	**MAP**	**S.E.**	**CI lower**	**CI upper**	p_{MAP}
Intercept	1.40	0.22	0.97	1.84	<.0001
Age	-0.71	0.08	-0.86	-0.56	<.0001
Education	0.11	0.07	-0.04	0.24	.341
Sex (male)	-0.30	0.05	-0.39	-0.20	<.0001
Age × Sex	-0.04	0.10	-0.24	0.16	.911
Age × Education	0.10	0.10	-0.10	0.30	.645
Sex × Education	-0.10	0.10	-0.29	0.09	.620
Perceptual speed	0.28	0.07	0.15	0.41	<.0001
Vocabulary	0.06	0.06	-0.06	0.18	.682
Verbal Fluency	0.07	0.06	-0.06	0.18	.629
TMT-B	-0.04	0.05	-0.14	0.06	.710
Episodic Memory	0.09	0.05	-0.02	0.18	.297
Recall	0.12	0.06	0.01	0.24	.118
Recognition	-0.01	0.05	-0.12	0.09	.973

Supplementary Table 15. Results of Bayesian multilevel binomial regression models predicting correct cued OID responses in incorrect free OID trials on the basis of demographic and cognitive variables, as well as free OID covariates included as control. The effects of recall and recognition are also shown in the table. Note that these results come from models where the Episodic memory variable had been replaced with these variables.

**Parameter**	**MAP**	**S.E.**	**CI lower**	**CI upper**	p_{MAP}
Intercept	1.02	0.21	0.61	1.46	<.0001
Age	-0.31	0.07	-0.44	-0.17	<.0001
Education	0.04	0.06	-0.09	0.16	.878
Sex (male)	-0.14	0.04	-0.22	-0.05	.008
Age × Sex	-0.04	0.09	-0.22	0.13	.884
Age × Education	0.05	0.09	-0.13	0.22	.848
Sex × Education	0.05	0.09	-0.12	0.21	.881
Perceptual speed	0.17	0.06	0.05	0.29	.016
Vocabulary	0.09	0.06	-0.02	0.20	.234
Verbal Fluency	-0.01	0.05	-0.12	0.09	.977
TMT-B	0.03	0.04	-0.06	0.11	.864
Episodic Memory	0.02	0.05	-0.07	0.11	.917
Recall	0.07	0.05	-0.03	0.18	.445
Recognition	-0.04	0.05	-0.13	0.06	.755
free OID covariate	0.8	0.05	0.7	0.89	<.0001

Supplementary Table 16. Results of Bayesian multilevel binomial regression models predicting correct cued OID responses in incorrect free OID trials on the basis of demographic and cognitive variables, without free OID covariates as control. The effects of recall and recognition are also shown in the table. Note that these results come from models where the Episodic memory variable had been replaced with these variables.

Parameter	MAP	S.E.	CI lower	CI upper	p_{MAP}
Intercept	1.08	0.21	0.63	1.47	<.0001
Age	-0.56	0.07	-0.69	-0.41	<.0001
Education	0.07	0.07	-0.06	0.20	.561
Sex (male)	-0.23	0.05	-0.33	-0.15	<.0001
Age × Sex	-0.04	0.10	-0.23	0.15	.918
Age × Education	0.06	0.10	-0.13	0.25	.807
Sex × Education	-0.04	0.09	-0.21	0.15	.984
Perceptual speed	0.24	0.06	0.12	0.37	<.0001
Vocabulary	0.07	0.06	-0.03	0.20	.377
Verbal Fluency	0.03	0.06	-0.08	0.14	.913
TMT-B	0.03	0.05	-0.06	0.12	.799
Episodic Memory	0.06	0.05	-0.03	0.16	.477
Recall	0.11	0.06	0.00	0.22	.170
Recognition	-0.02	0.05	-0.12	0.07	.893

Supplementary Table 17. Results of Bayesian multilevel binomial regression models predicting correct cued OID responses on the basis of demographic and cognitive variables, free OID covariates, and age interaction effects.

Parameter	MAP	S.E.	Lower	Upper	p_{MAP}
Intercept	1.34	0.24	0.88	1.82	<.0001
Age	-0.21	0.05	-0.31	-0.12	<.0001
Sex (male)	-0.09	0.03	-0.15	-0.03	.008
Education	0.01	0.03	-0.05	0.08	.928
Perceptual speed	0.07	0.04	-0.02	0.14	.390
Vocabulary	-0.04	0.04	-0.11	0.04	.665
Verbal Fluency	0.01	0.04	-0.07	0.08	.993
TMT-B	0.02	0.03	-0.05	0.09	.824
Episodic Memory	0.05	0.03	-0.01	0.12	.299
Free OID covariate	2.09	0.03	2.02	2.16	<.0001
Age × Sex	0.02	0.06	-0.10	0.15	.946
Age × Education	0.04	0.07	-0.11	0.17	.879
Age × Perceptual speed	0.02	0.08	-0.13	0.18	.951
Age × Vocabulary	0.03	0.08	-0.13	0.17	.916
Age × Verbal Fluency	0.01	0.08	-0.16	0.16	.997
Age × TMT-B	-0.08	0.06	-0.19	0.04	.406
Age × Episodic Memory	-0.03	0.07	-0.16	0.09	.890
Age × Free OID covariate	-0.12	0.07	-0.25	0.02	.283

Supplementary Table 18. Results of Bayesian multilevel multinomial regression model with omissions as reference level, predicting correct and incorrect free OID responses on the basis of demographic and cognitive variables, as well as cued OID covariates included as control. The effects of recall and recognition are also shown in the table. Note that these results come from models where the Episodic memory variable had been replaced with these variables.

**Comp.**	**Parameter**	**MAP**	**S.E.**	**Lower CI**	**Upper CI**	p_{MAP}
**Correct**	Intercept	-1.06	0.27	-1.60	-0.54	.002
	Age	-0.74	0.10	-0.92	-0.54	<.0001
	Sex (male)	-0.44	0.06	-0.57	-0.32	<.0001
	Education	0.20	0.09	0.03	0.36	.072
	Age × Sex	-0.23	0.13	-0.49	0.03	.231
	Age × Education	0.12	0.13	-0.12	0.40	.607
	Sex × Education	-0.32	0.12	-0.55	-0.08	.026
	Perceptual speed	0.19	0.08	0.03	0.35	.062
	Vocabulary	-0.20	0.08	-0.36	-0.04	.052
	Verbal fluency	0.24	0.08	0.10	0.40	.005
	TMT-B	-0.02	0.07	-0.15	0.11	.961
	Episodic memory	0.23	0.07	0.10	0.35	.002
	Recall	0.12	0.07	-0.02	0.27	.255
	Recognition	0.15	0.07	0.01	0.27	.135
	cued OID covariate	1.05	0.07	0.92	1.18	<.0001
**Incorrect**	Intercept	-0.14	0.13	-0.39	0.12	.519
	Age	-0.22	0.09	-0.39	-0.04	.049
	Sex (male)	0.15	0.08	0.00	0.32	.147
	Education	-0.41	0.06	-0.52	-0.30	<.0001
	Age × Sex	-0.28	0.12	-0.53	-0.06	.050
	Age × Education	0.09	0.12	-0.15	0.31	.792
	Sex × Education	-0.12	0.11	-0.32	0.11	.661
	Perceptual speed	0.04	0.08	-0.10	0.20	.795
	Vocabulary	-0.27	0.07	-0.40	-0.11	.002
	Verbal fluency	0.25	0.07	0.11	0.38	.003
	TMT-B	0.02	0.06	-0.10	0.13	.970
	Episodic Memory	0.18	0.06	0.07	0.31	.006
	Recall	0.07	0.07	-0.06	0.21	.593
	Recognition	0.14	0.06	0.01	0.26	.079
	cued OID covariate	0.35	0.06	0.24	0.47	<.0001

Supplementary Table 19. Results of Bayesian multilevel multinomial regression model with omissions as reference level, predicting correct and incorrect free OID responses on the basis of demographic and cognitive variables, without cued OID covariates included as control. The effects of recall and recognition are also shown in the table. Note that these results come from models where the Episodic memory variable had been replaced with these variables.

**Comp.**	**Parameter**	**MAP**	**S.E.**	**Lower CI**	**Upper CI**	$p_{\mathbf{MAP}}$
**Correct**	Intercept	-1.01	0.27	-1.51	-0.46	.002
	Age	-0.98	0.10	-1.18	-0.78	<.0001
	Sex (male)	0.23	0.09	0.05	0.40	.054
	Education	-0.54	0.06	-0.67	-0.42	<.0001
	Age × Sex	-0.24	0.14	-0.53	0.02	.197
	Age × Education	0.17	0.14	-0.09	0.46	.432
	Sex × Education	-0.33	0.12	-0.58	-0.09	.030
	Perceptual speed	0.30	0.09	0.13	0.47	.002
	Vocabulary	-0.15	0.08	-0.31	0.02	.219
	Verbal fluency	0.26	0.08	0.11	0.42	.004
	TMT-B	-0.03	0.07	-0.17	0.10	.881
	Episodic memory	0.25	0.07	0.11	0.38	.001
	Recall	0.17	0.08	0.02	0.32	.094
	Recognition	0.12	0.07	-0.01	0.26	.210
**Incorrect**	Intercept	-0.12	0.13	-0.38	0.14	.629
	Age	-0.32	0.09	-0.48	-0.14	.002
	Sex (male)	0.18	0.08	0.01	0.33	.113
	Education	-0.45	0.06	-0.56	-0.34	<.0001
	Age × Sex	-0.30	0.12	-0.54	-0.06	.058
	Age × Education	0.10	0.12	-0.14	0.33	.729
	Sex × Education	-0.12	0.11	-0.32	0.12	.676
	Perceptual speed	0.08	0.08	-0.07	0.24	.515
	Vocabulary	-0.24	0.07	-0.39	-0.10	.005
	Verbal fluency	0.25	0.07	0.12	0.39	.002
	TMT-B	0.00	0.06	-0.11	0.12	1.000
	Episodic memory	0.20	0.06	0.08	0.31	.007
	Recall	0.07	0.07	-0.05	0.22	.491
	Recognition	0.14	0.06	0.02	0.26	.089

Supplementary Table 20. Results of Bayesian multilevel multinomial regression model with incorrect responses as reference level, predicting omissions and correct free OID responses on the basis of demographic and cognitive variables, as well as cued OID covariates included as control. The effects of recall and recognition are also shown in the table. Note that these results come from models where the Episodic memory variable had been replaced with these variables.

**Comp.**	**Parameter**	**MAP**	**S.E.**	**Lower CI**	**Upper CI**	p_{MAP}
**Correct**	Intercept	-0.90	0.25	-1.42	-0.41	.005
	Age	-0.51	0.07	-0.65	-0.36	<.0001
	Sex (male)	-0.05	0.05	-0.15	0.04	.509
	Education	0.04	0.06	-0.09	0.17	.797
	Age × Sex	0.01	0.11	-0.20	0.22	.995
	Age × Education	0.10	0.10	-0.12	0.29	.718
	Sex × Education	-0.20	0.09	-0.38	-0.03	.081
	Perceptual speed	0.14	0.06	0.03	0.27	.058
	Vocabulary	0.05	0.06	-0.07	0.17	.704
	Verbal fluency	0.01	0.06	-0.10	0.13	.963
	TMT-B	-0.02	0.05	-0.12	0.08	.897
	Episodic memory	0.06	0.05	-0.05	0.15	.573
	Recall	0.07	0.06	-0.04	0.18	.450
	Recognition	0.00	0.05	-0.11	0.1	.998
	cued OID covariate	0.68	0.05	0.57	0.77	<.0001
**Omission**	Intercept	0.03	0.13	-0.23	0.30	.953
	Age	0.24	0.10	0.04	0.43	.054
	Sex (male)	-0.18	0.09	-0.36	-0.01	.112
	Education	0.44	0.06	0.32	0.56	<.0001
	Age × Sex	0.30	0.13	0.05	0.57	.064
	Age × Education	-0.07	0.13	-0.33	0.19	.883
	Sex × Education	0.17	0.12	-0.09	0.39	.471
	Perceptual speed	-0.05	0.08	-0.21	0.12	.829
	Vocabulary	0.28	0.08	0.12	0.44	.004
	Verbal fluency	-0.27	0.08	-0.42	-0.12	.002
	TMT-B	-0.02	0.07	-0.14	0.11	.977
	Episodic memory	-0.20	0.07	-0.33	-0.08	.007
	Recall	-0.08	0.08	-0.23	0.06	.574
	Recognition	-0.15	0.07	-0.29	-0.02	.091
	cued OID covariate	-0.39	0.06	-0.52	-0.27	<.0001

Supplementary Table 21. Results of Bayesian multilevel multinomial regression model with incorrect responses as reference level, predicting omissions and correct free OID responses on the basis of demographic and cognitive variables, without cued OID covariates included as control. The effects of recall and recognition are also shown in the table. Note that these results come from models where the Episodic memory variable had been replaced with these variables.

Comp.	Parameter	MAP	S.E.	Lower CI	Upper CI	p_{MAP}
Correct	Intercept	-0.89	0.24	-1.35	-0.37	.007
	Age	-0.65	0.08	-0.80	-0.50	<.0001
	Sex (male)	0.05	0.07	-0.07	0.19	.667
	Education	-0.11	0.05	-0.21	-0.01	.095
	Age × Sex	-0.01	0.11	-0.22	0.22	.997
	Age × Education	0.12	0.11	-0.10	0.33	.568
	Sex × Education	-0.21	0.09	-0.40	-0.04	.078
	Perceptual speed	0.21	0.07	0.09	0.34	.007
	Vocabulary	0.08	0.06	-0.04	0.21	.478
	Verbal fluency	0.03	0.06	-0.10	0.14	.925
	TMT-B	-0.03	0.05	-0.14	0.07	.852
	Episodic memory	0.06	0.05	-0.04	0.17	.468
	Recall	0.10	0.06	-0.02	0.21	.260
	Recognition	-0.02	0.05	-0.12	0.09	.960
Omission	Intercept	0.01	0.14	-0.24	0.30	.987
	Age	0.35	0.10	0.15	0.54	.003
	Sex (male)	-0.21	0.09	-0.37	-0.02	.091
	Education	0.47	0.06	0.35	0.60	<.0001
	Age × Sex	0.33	0.13	0.06	0.58	.067
	Age × Education	-0.10	0.13	-0.35	0.17	.794
	Sex × Education	0.13	0.12	-0.10	0.39	.468
	Perceptual speed	-0.08	0.09	-0.27	0.07	.559
	Vocabulary	0.26	0.08	0.10	0.42	.006
	Verbal fluency	-0.28	0.08	-0.43	-0.12	.003
	TMT-B	0.00	0.07	-0.13	0.13	.999
	Episodic memory	-0.21	0.07	-0.35	-0.08	.008
	Recall	-0.10	0.08	-0.25	0.06	.491
	Recognition	-0.15	0.07	-0.28	-0.01	.110

Supplementary Table 22. Results of Bayesian multilevel multinomial regression model with correct responses as reference level, predicting omissions and incorrect free OID responses on the basis of demographic and cognitive variables, as well as cued OID covariates included as control. The effects of recall and recognition are also shown in the table. Note that these results come from models where the Episodic memory variable had been replaced with these variables.

Comp.	Parameter	MAP	S.E.	Lower CI	Upper CI	p_{MAP}
**Omission**	Intercept	0.88	0.27	0.36	1.42	.007
	Age	0.72	0.10	0.51	0.92	<.0001
	Sex (male)	0.48	0.07	0.35	0.61	<.0001
	Education	-0.22	0.09	-0.42	-0.05	.054
	Age × Sex	0.28	0.14	0.02	0.58	.131
	Age × Education	-0.15	0.14	-0.44	0.12	.542
	Sex × Education	0.34	0.13	0.09	0.59	.026
	Perceptual speed	-0.19	0.09	-0.37	-0.02	.106
	Vocabulary	0.23	0.09	0.06	0.39	.038
	Verbal fluency	-0.29	0.08	-0.44	-0.13	.002
	TMT-B	0.01	0.07	-0.12	0.15	.985
	Episodic memory	-0.25	0.07	-0.39	-0.11	.001
	Recall	-0.15	0.08	-0.31	0.01	.178
	Recognition	-0.13	0.07	-0.28	0.01	.174
	cued OID covariate	-1.01	0.07	-1.15	-0.88	<.0001
**Incorrect**	Intercept	0.81	0.24	0.33	1.30	.006
	Age	0.47	0.07	0.33	0.61	<.0001
	Sex (male)	-0.04	0.06	-0.16	0.09	.818
	Education	0.03	0.05	-0.06	0.13	.761
	Age × Sex	-0.04	0.11	-0.24	0.16	.930
	Age × Education	-0.07	0.10	-0.27	0.13	.769
	Sex × Education	0.21	0.09	0.03	0.37	.077
	Perceptual speed	-0.13	0.06	-0.26	-0.01	.086
	Vocabulary	-0.07	0.06	-0.18	0.05	.549
	Verbal fluency	-0.01	0.06	-0.12	0.10	.985
	TMT-B	0.03	0.05	-0.07	0.13	.864
	Episodic memory	-0.03	0.05	-0.13	0.06	.726
	Recall	-0.07	0.06	-0.18	0.04	.473
	Recognition	0.02	0.05	-0.08	0.12	.934
	cued OID covariate	-0.61	0.05	-0.71	-0.51	<.0001

Supplementary Table 23. Results of Bayesian multilevel multinomial regression model with correct responses as reference level, predicting omissions and incorrect free OID responses on the basis of demographic and cognitive variables, without cued OID covariates included as control. The effects of recall and recognition are also shown in the table. Note that these results come from models where the Episodic memory variable had been replaced with these variables.

Comp.	Parameter	MAP	S.E.	Lower CI	Upper CI	p_{MAP}
**Omission**	Intercept	0.78	0.26	0.28	1.33	.013
	Age	0.94	0.11	0.73	1.15	<.0001
	Sex (male)	-0.25	0.09	-0.44	-0.07	.028
	Education	0.57	0.07	0.43	0.70	<.0001
	Age × Sex	0.32	0.15	0.02	0.60	.115
	Age × Education	-0.21	0.14	-0.48	0.09	.397
	Sex × Education	0.36	0.13	0.10	0.61	.025
	Perceptual speed	-0.28	0.09	-0.46	-0.11	.006
	Vocabulary	0.17	0.09	0.01	0.35	.129
	Verbal fluency	-0.29	0.08	-0.46	-0.14	.001
	TMT-B	0.03	0.07	-0.11	0.17	.892
	Episodic memory	-0.26	0.07	-0.40	-0.12	.001
	Recall	-0.19	0.08	-0.34	-0.02	0.073
	Recognition	-0.13	0.07	-0.27	0.02	0.243
**Incorrect**	Intercept	0.77	0.24	0.27	1.23	.009
	Age	0.59	0.07	0.45	0.73	<.0001
	Sex (male)	-0.06	0.06	-0.18	0.07	.718
	Education	0.08	0.05	-0.02	0.17	.262
	Age × Sex	-0.02	0.11	-0.23	0.18	.969
	Age × Education	-0.10	0.10	-0.30	0.11	.658
	Sex × Education	0.21	0.09	0.03	0.38	.069
	Perceptual speed	-0.18	0.06	-0.31	-0.06	.014
	Vocabulary	-0.09	0.06	-0.21	0.03	.318
	Verbal fluency	-0.02	0.06	-0.13	0.10	.936
	TMT-B	0.03	0.05	-0.06	0.14	.744
	Episodic memory	-0.04	0.05	-0.14	0.05	.653
	Recall	-0.10	0.06	-0.20	0.02	.274
	Recognition	0.03	0.05	-0.07	0.13	.839

4. Posterior predictive checks

In order to evaluate the Bayesian models' predictive ability, we performed posterior predictive checks on the binomial and the multinomial models. This involves drawing simulated data based on the model parameters, and then comparing those simulations to the observed data (Gelman et al. 1996). The goodness-of-fit between some statistic based on the observed data and the same statistic based on the simulated data provides an illustration of the models' predictive ability. Here, we investigated the by-odor percentage of each response type (correct free OID, misnaming, omission and identification). From each model, we drew 1000 simulated data sets. On the basis of these, we calculated the 95% lower and upper percentages of each response type for each odor (e.g., the percentage of omissions for the apple odor). In order to illustrate the models' predictive ability of a single draw, we also obtained the by-odor percentages from the first of each of the 1000 simulated data sets of each model. This was done both for the binomial models, on the one hand, and the multinomial models, on the other. The results of these analyses, in terms of the correlations between the predicted and observed by-odor percentages, are illustrated in Supplementary Figure 1 (binomial models) and Supplementary Figure 2 (multinomial models).

In the figures, gray areas illustrate the 95% lower and upper by-odor percentages, and the dotted lines show the fit between observed percentages. Deviations from this line on the y-axis thus illustrate deviations between predicted and observed by-odor percentages, which overall are low. Crucially, the 95% lower and upper predicted by-odor percentages capture the observed by-odor percentages well, attesting to high predictive ability of all models.

5. Hierarchical regression models of OID scores

For comparability with earlier studies (e.g., Larsson et al. 2004), we analyzed the relationships between the demographic and cognitive variables, on the one hand, and free and cued OID covariates, on the other, with hierarchical regression modeling. As in previous studies, analyses were conducted on participant-level data (i.e., one data point per participant). Rows with missing data were excluded before analyses were run. Model 1 only included the demographic variables Age, Education and Sex. Model 2 also included their 1-way interaction effects. In Model 3, all cognitive variables were added. And in model 4, finally, the control OID score—cued OID covariates in the analysis of free OID covariates, and free OID covariates in the analysis of cued OID covariates—was added. The results of this analysis are shown in Supplementary Table 24. We also ran separate models that included separate recall and recognition score. The effects of these variables are included in Supplementary Table 24.

The results of the model 4 models of both free and cued OID covariates are overall highly similar to those of the multilevel Bayesian models. Both free and cued OID covariates are negatively associated with age and worse for men. free OID covariates are also negatively associated with education level for men. Whereas free OID covariates are positively associated with Perceptual speed, Verbal fluency and Episodic memory, cued OID covariates only show a positive association with Perceptual speed. This confirms the general conclusion of the multilevel analyses that free OID is more cognitively demanding than cued OID in terms of drawing on a broader set of cognitive abilities.

Comparisons between model 3 and the model 4 models show, also in line with the results of the multilevel models, that the inclusion of the other type of OID score as control mainly had a weakening effect on the associations between age and sex, on the one hand, and the OID score at

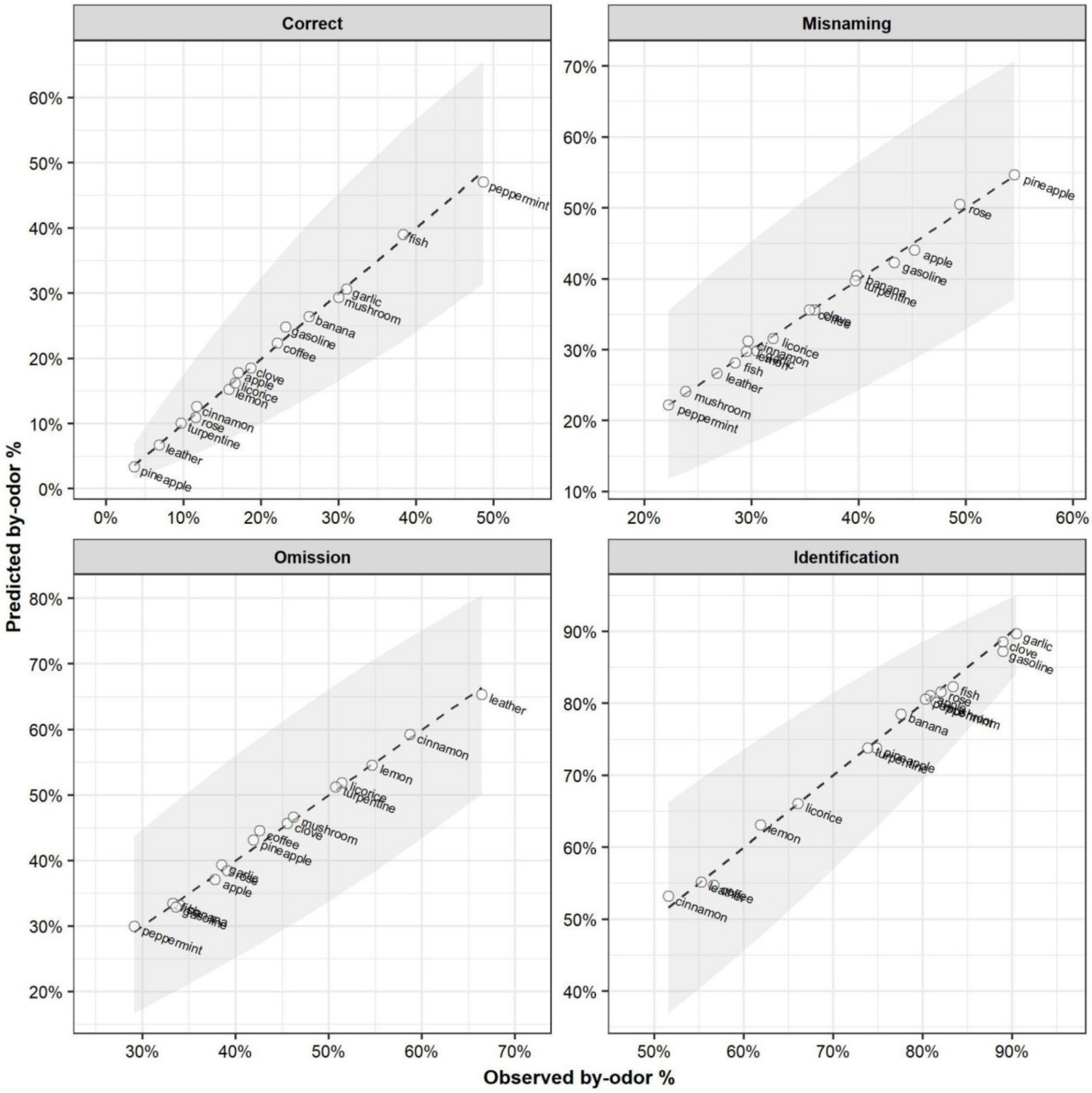

Supplementary Figure 1. Posterior predictive checks of binomial models predicting correct free OIDs, misnamings, omissions and correct cued OIDs in terms of predicted by-odor percentages, calculated on the basis from the first model simulation, as a function of observed by-odor percentages, calculated on the basis of the observed data. Shaded areas illustrate 95% lower and upper percentage intervals, calculated on the basis of 1000 model simulation draws.

hand, on the other. This is also the case for perceptual speed, to some degree. These findings indicate that age, sex and perceptual speed are important factors in both free OID / odor naming and cued OID.

Comparisons between model 2 and model 3 models further show that the inclusion of the cognitive variables had a weakening effect on the associations between age and education, on the one hand, and OID scores, on the other. This finding reflects the associations between these demographic variables and the cognitive variables: whereas education length is positively associated with cognitive abilities, most likely because higher eduction is cognitively demanding, age and cognitive abilities are negatively associated due to age-related cognitive decline.

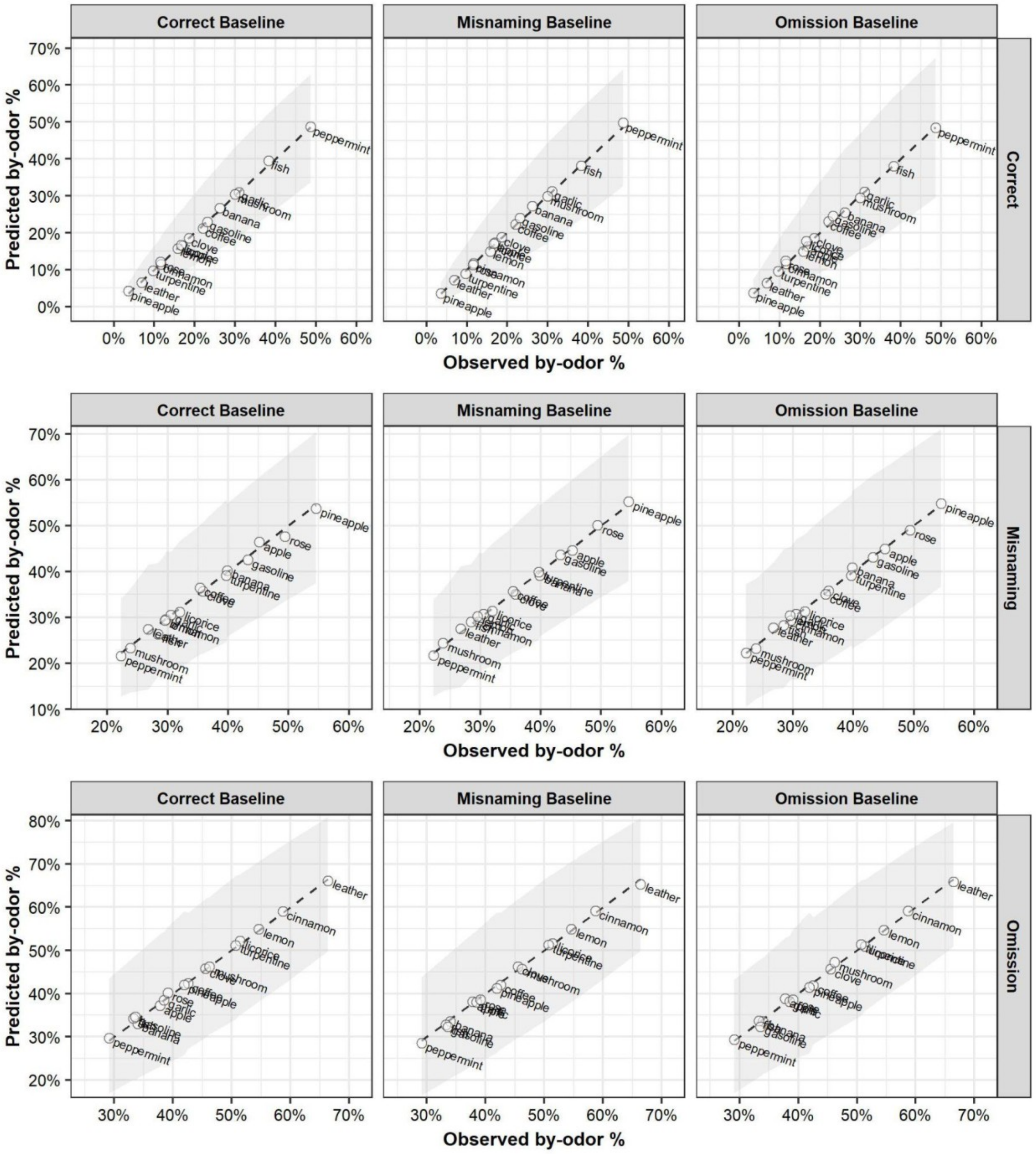

Supplementary Figure 2. Posterior predictive checks of multinomial models, differentiated by baseline category (columns), predicting correct free OIDs, misnamings, and omissions (rows) in terms of predicted by-odor percentages, calculated on the basis from the first model simulation, as a function of observed by-odor percentages, calculated on the basis of the observed data. Shaded areas illustrate 95% lower and upper percentage intervals, calculated on the basis of 1000 model simulation draws.

A comparison between free OID model 1 and free OID model 2 highlights an interesting finding: whereas the association between free OID covariate and eduction level is positive for women, it is negative for men, as clearly illustrated by the fact that the positive association between education and free OID covariate is strengthened by the inclusion of the eduction × sex interaction. For men, the association is in the opposite direction. In other words, whereas highly educated women are better at odor naming than women with low education, highly educated men perform worse than men with lower education.

Supplementary Table 24. Results of hierarchical regression modeling of free and cued OID covariates, performed on the level of each participant. Model 1: demographic variables; model 2: demographic variables + demographic interaction effects; model 3: cognitive variables; total or free OID covariates as control variable. The effects of recall and recognition are also shown in the table. Note that these results come from models where the Episodic memory variable had been replaced with these variables.

OID type	Coefficient	Model 1	Model 2	Model 3	Model 4
**Free**	(Intercept)	0.07***	0.07***	0.06***	0.05***
	Age	-0.43***	-0.43***	-0.34***	-0.26***
	Education	0.05*	0.10**	0.06*	0.05
	Sex (male)	-0.16***	-0.15***	-0.13***	-0.10***
	Age × Sex		0.01	0.00	0.01
	Age × Education		0.03	0.03	0.01
	Education × Sex		-0.11*	-0.12**	-0.12**
	Perceptual speed			0.09**	0.06*
	Vocabulary			-0.02	-0.03
	Verbal fluency			0.07**	0.06*
	TMT-B			0.01	0.00
	Episodic memory			0.06**	0.05*
	Recall			0.06*	0.05*
	Recognition			0.01	0.02
	Cued OID score				0.31***
	R-square	0.187	0.190	0.205	0.281
	Adjusted r-square	0.185	0.187	0.201	0.276
**Cued**	(Intercept)	0.05***	0.06***	0.05***	0.03*
	Age	-0.35***	-0.34***	-0.25***	-0.15***
	Education	0.06**	0.06*	0.02	0.00
	Sex (male)	-0.10*	-0.11***	-0.10***	-0.06**
	Age × Sex		-0.02	-0.02	-0.02
	Age × Education		0.07	0.06	0.05
	Education × Sex		0.01	0.00	0.04
	Processing speed			0.11***	0.08**
	Vocabulary			0.04	0.05
	Verbal fluency			0.02	0.00
	TMT-B			0.02	0.02
	Episodic memory			0.02	0.00
	Recall			0.04	0.02
	Recognition			-0.01	-0.01
	free OID covariate				0.31***
	R-square	0.131	0.132	0.146	0.227
	Adjusted r-square	0.130	0.130	0.141	0.222

*: $p < .05$; **: $p < .01$; ***: $p < .0001$

A final observation that should be mentioned concerns the differences in r^2 between the models. Comparing models with respect to r^2 shows that it is mainly the demographic variables and the opposite OID scores that account for the bulk of the variance in the models. In other words, the main predictors of OID performance are age and sex, and free and cued OID are highly related tasks.

References

Larsson, M., Nilsson, L. G., Olofsson, J. K., & Nordin, S. (2004). Demographic and Cognitive Predictors of Cued Odor Identification: Evidence from a Population-based Study. Chemical Senses, 29(6), 547–554. https://doi.org/10.1093/chemse/bjh059

Gelman, A., Meng, X.-L., & Stern, H. (1996). Posterior Predictive Assessment of Model Fitness Via Realized Discrepancies. *Statistica Sinica*, *6*(4), 733–760.
